# Development of a radiographic scoring system for new bone formation in gout

**DOI:** 10.1186/s13075-021-02683-9

**Published:** 2021-12-08

**Authors:** Chang-Nam Son, Ken Cai, Sarah Stewart, John Ferrier, Karen Billington, Yun-Jung Jack Tsai, Thomas Bardin, Anne Horne, Lisa K. Stamp, Anthony Doyle, Nicola Dalbeth

**Affiliations:** 1 Keimyung University School of Medicine, Daegu, South Korea; 2 Department of Medicine, Faculty of Medical and Health Sciences, The University of Auckland, Auckland, New Zealand; 3 Department of Radiology, Auckland District Health Board, Auckland, New Zealand; 4 Department of Rheumatology, Hôpital Lariboisière, Paris, France; 5 Department of Medicine, University of Otago Christchurch, Christchurch, New Zealand; 6 Department of Radiology with Anatomy, Faculty of Medical and Health Sciences, The University of Auckland, Auckland, New Zealand

**Keywords:** Gout, New bone formation, Radiography

## Abstract

**Background:**

Features of new bone formation (NBF) are common in tophaceous gout. The aim of this project was to develop a plain radiographic scoring system for NBF in gout.

**Methods:**

Informed by a literature review, scoring systems were tested in 80 individual 1st and 5th metatarsophalangeal joints. Plain radiography scores were compared with computed tomography (CT) measurements of the same joints. The best-performing scoring system was then tested in paired sets of hand and foot radiographs obtained over 2 years from an additional 25 patients. Inter-reader reproducibility was assessed using intraclass correlation coefficients (ICC). NBF scores were correlated with plain radiographic erosion scores (using the gout-modified Sharp-van der Heijde system).

**Results:**

Following a series of structured reviews of plain radiographs and scoring exercises, a semi-quantitative scoring system for sclerosis and spur was developed. In the individual joint analysis, the inter-observer ICC (95% CI) was 0.84 (0.76–0.89) for sclerosis and 0.81 (0.72–0.87) for spur. Plain radiographic sclerosis and spur scores correlated with CT measurements (*r* = 0.65–0.74, *P* < 0.001 for all analyses). For the hand and foot radiograph sets, the inter-observer ICC (95% CI) was 0.94 (0.90–0.98) for sclerosis score and 0.76 (0.65–0.84) for spur score. Sclerosis and spur scores correlated highly with plain radiographic erosion scores (*r* = 0.87 and 0.71 respectively), but not with change in erosion scores over 2 years (*r* = −0.04–0.15).

**Conclusion:**

A semi-quantitative plain radiographic scoring method for the assessment of NBF in gout is feasible, valid, and reproducible. This method may facilitate consistent measurement of NBF in gout.

**Supplementary Information:**

The online version contains supplementary material available at 10.1186/s13075-021-02683-9.

## Introduction

Features of new bone formation such as sclerosis and spurs are common on plain radiography in tophaceous gout [1]. While a plain radiographic damage scoring system incorporating bone erosion and joint space narrowing has been developed [2], there are no published methods for scoring new bone formation in gout.

Although there have been some reports of reparative processes with increased new bone formation in response to intensive urate-lowering therapy [3, 4], it is not possible to systematically quantify these changes at present, due to the lack of a validated scoring method. The aim of this project was to develop a plain radiographic scoring system for new bone formation in patients with gout.

## Materials and methods

Plain radiographs and computed tomography (CT) scans were obtained for research, approved by the New Zealand Ministry of Health (NTX/08/06/050/AM06 and MEC/11/06/060). The patients were diagnosed on the basis of the 2015 American College of Rheumatology/European League Against Rheumatism gout classification criteria [5]. All participants provided written informed consent. Previously published definitions of new bone formation features in gout were used in this project [1]: ankylosis—fusion of the bones of a joint, with trabeculae crossing the joint space; osteophyte—bone projection arising along the joint margin and associated with cartilage; periosteal new bone formation—bone proliferation arising from the periosteum; sclerosis—increased density of medullary or subcortical bone; and spur—a sharp spicule of dense bone proliferation extending at an acute angle from the cortex.

### Literature review of scoring systems

We initially performed a scoping literature review of scoring systems for new bone formation in bone and joint diseases, searching in MEDLINE (via PubMed) and EMBASE databases, published from January 1, 1946, to February 28, 2020. The first search was performed on April 29, 2019, and repeated on February 28, 2020, to capture any additional studies published since the original search was performed. Details of each unique scoring system and the numbers of papers reporting each scoring system were extracted (Supplementary Table 1). The protocol was registered on PROSPERO (CRD: 42019138077; 28-08-2019).

### Initial testing of new bone formation scoring system in individual joints

Two rheumatologists (KC and ND) and a musculoskeletal (MSK) radiologist (AD) undertook a structured review of plain radiographs from patients with gout. Informed by the literature review, a range of scoring systems including semi-quantitative scoring systems used in osteoarthritis (Oslo Hand Osteoarthritis MRI Score [6] and Boston Leeds Osteoarthritis Knee Score [7]) and psoriatic arthritis (proliferation score of the Psoriatic Arthritis Ratingen Score (PARS) [8]) were chosen for scoring (Supplementary Table 2). In addition, quantitative measurement of lesions in millimeters and the ratio of the length of the largest lesion relative to the width of the adjacent joint surface in millimeters was selected. In an inter-reader reproducibility exercise, a rheumatologist (KC) and MSK radiologist (JF) scored 80 individual joints (40 1st metatarsophalangeal (MTP) joints and 40 5th MTP joints) from 20 separate patients with gout using these methods (see Supplementary Table 3 for clinical features of these participants).

### Refinement and retesting of new bone formation scoring system in individual joints

**Fig. 1:**
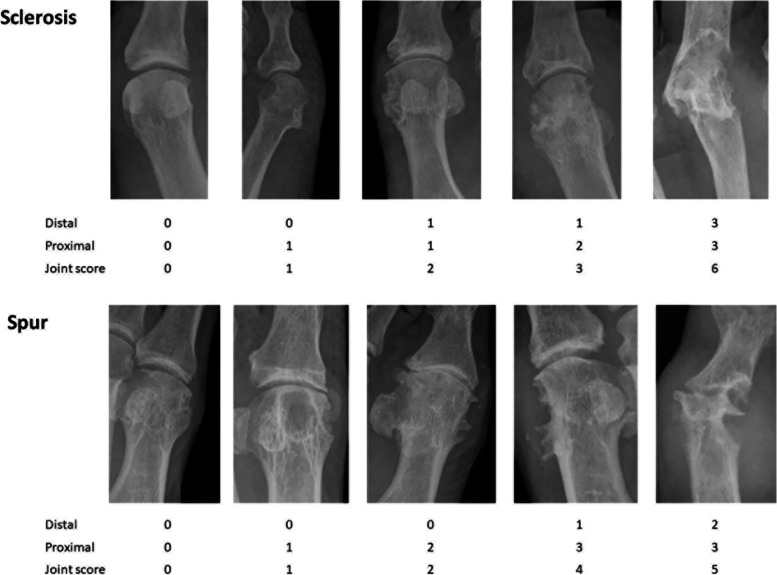
Examples of sclerosis and spur scores at individual joints

Following these initial results, we proceeded to a second structured review of a new set of plain radiographs (AD, KC, and ND). This work included standardization of definitions, approaches, and projections and development of reference images for scoring (Fig. 1). In a further inter-reader reproducibility exercise, two rheumatologists (CS and ND) rescored the same 80 individual joints (40 1st MTP joints and 40 5th MTP joints) for sclerosis and spur using the new scoring system, modified from the Kannus semi-quantitative plain radiographic scoring system for sclerosis in knee osteoarthritis [9] and the Thumb Base Osteoarthritis Magnetic Resonance Imaging Scoring System (TOMS) respectively [10].

### Comparison with CT measurements of individual joints

Construct validity was assessed by correlating the plain radiography scores with CT measurements of sclerosis and spur at the same individual joints, as previously described [1]. CT is considered the reference standard for the assessment of bone structural changes in arthritis [11, 12] and allows measurement in three dimensions. A MSK radiologist (YJT), who was blinded to all plain radiographic scoring, analyzed the CT scans obtained on the same date as the plain radiographs. The following sclerosis data were measured at the same 80 individual joints (40 1st MTP joints and 40 5th MTP joints): the presence of sclerosis, Hounsfield units at the site of maximum sclerosis, and the ratio of the depth of sclerosis relative to the width of the adjacent joint surface (in any dimension). The following spur data were measured at the same joints: the presence of spur, length of the largest spur in millimeters (in any dimension), and the ratio of the depth of sclerosis relative to the width of the adjacent joint surface (in any dimension).

### Testing of scoring system in hand and foot radiographs

The scoring system was then tested in paired sets of hand and foot radiographs over a 2-year period from a further 25 patients with gout receiving treat to serum urate target (< 0.36mmol/L) gout management (at baseline, mean (standard deviation (*SD*)) disease duration 22 (11) years, 72% with subcutaneous tophi, mean (*SD*) allopurinol dose at baseline 296 (149) mg/day, with dose titration to 430 (185) mg/day at year 2, serum urate 0.43 (0.11) mmol/L at baseline, reducing to 0.32 (0.06) mmol/L at year 2; see Supplementary Table 4 for detailed clinical characteristics of these participants). In an inter-reader reproducibility exercise, the hand and foot radiographs were scored by two rheumatologists (CS and ND), using the same regions scored in the gout-modified Sharp-van der Heijde scoring system for bone erosion [2, 13] (hand distal interphalangeal joints, proximal interphalangeal joints, interphalangeal (IP) joint of the thumb, metacarpophalangeal joints, base of the first metacarpal, multangular, scaphoid, lunate, radius, ulna, MTP joints, and IP joints of the hallux). The proximal and distal bones were scored separately for joints with long bones (maximum score 6 for each joint), and single bones for the carpal and tarsal bones (maximum score 3 for each bone). The same paired sets of hand and foot radiographs were scored for the gout-modified Sharp-van der Heijde erosion scores [2, 13] by a MSK radiologist (KB). All readers were blinded to each other’s scores. Paired sets of radiographs were scored in a known order.

### Statistical analysis

Inter-reader reliability was assessed by intraclass correlation coefficients (ICCs) with 95% confidence intervals (95% CI). Median and interquartile range (IQR) was used to describe new bone formation scores. Correlations between the new bone formation scores (average of two scorers) and other modalities/scoring systems as well as clinical characteristics were assessed using Spearman’s correlation coefficients. Differences between the groups were analyzed using the Mann-Whitney *U* and Wilcoxon signed-rank tests. All statistical analyses were performed using SPSS (version 25.0, IBM Corp., Armonk, NY, USA).

## Results

### Literature review of scoring systems

There were 840 papers identified, reporting 86 unique scoring systems for new bone formation (Supplementary Fig. S1). These scoring systems were applicable to a range of musculoskeletal conditions including osteoarthritis (*n* = 61 scoring systems), spondyloarthropathies (*n* = 16), diffuse idiopathic skeletal hyperostosis (*n* = 2), heterotopic ossification (*n* = 2), rheumatoid arthritis (*n* = 2), psoriatic arthritis (*n* = 1), slipped capital femoral epiphysis (*n* = 1), and juvenile idiopathic arthritis (*n* = 1). New bone formation was scored either as part of a composite score (*n* = 40 unique scoring systems) or as a stand-alone score (*n* = 44) or a combination of both composite and stand-alone scoring systems (*n* = 2). Those involving assessment of the individual features of new bone formation used semi-quantitative scales (i.e., none, mild, medium, severe) (*n* = 41, 89%). No scoring system used a quantitative scale. The most commonly used scoring systems are shown in Supplementary Table 1.

### Initial testing of new bone formation scoring system in individual joints

Following the first structured review of plain radiographs, 80 MTP joints were scored by two readers based on osteoarthritis and psoriatic scoring systems, as well as quantitative measurement of lesions (Supplementary Table 2). Inter-reader ICCs were low (0.23–0.64 for osteophyte, 0.35–0.54 for sclerosis, and − 0.05 to 0.39 for spur) (Supplementary Table 5). No periosteal bone formation was scored by either reader, and one joint was scored for ankylosis by both readers.

### Refinement and retesting of the new bone formation scoring system in individual joints

In the second structured review of plain radiographs, definitions, approaches, and projections for scoring were discussed and standardized. As sclerosis and spurs were the most common lesions specific to gout, we focused on developing a scoring system for these features, noting that although osteophytes are common in joints affected by gout, they are non-specific. Additionally, a scoring system for periosteal new bone formation and ankylosis was not progressed as these lesions were so uncommon. The low inter-reader reproducibility using the PARS system also led us to develop separate scores for sclerosis and spur, rather than a single new bone formation score.

The Kannus semi-quantitative plain radiographic scoring system for sclerosis in knee osteoarthritis [9] was modified for use in gout, as follows: 0 = no sclerosis, 1 = < 1/3 bone affected, 2 = 1/3–2/3 bone affected, and 3 = > 2/3 bone affected. The following rules were agreed for MTP joints: the proximal and distal bones were scored separately and summed for a total sclerosis score (maximum score 6 for each joint), the bone within 1-cm distance from the articular surface was scored, and if the bone was eroded, available bone was used as the denominator. Only the anterior-posterior view was used to assess sclerosis.

A key challenge for spur scoring was that joints within the gout scoring system have variable size and affected bones could have more than one spur. For this reason, the Thumb Base Osteoarthritis Magnetic Resonance Imaging Scoring System [10] was modified for scoring spurs in gout; this system captured both spur size and number: 0 = no spurs, 1 = 1 small spur, 2 = ≥ 2 small spurs and/or 1 moderate spur, and 3 = ≥ 2 moderate spurs and/or ≥ 1 large spur(s). As with sclerosis, the proximal and distal bones were scored for spur separately and summed for a total spur score, and the bone within a 1-cm distance from the articular surface was scored. Both the anterior-posterior and oblique views were used to assess spurs.

The standardized definitions and projections are shown in Table 1, and examples of the scoring system are shown in Fig. 1. Rescoring of 80 individual joints (40 1st MTP joints and 40 5th MTP joints) for sclerosis and spur using this new scoring system and associated rules led to an improved inter-reader agreement: for sclerosis 0.84 (0.76–0.89) and for spur 0.81 (0.72–0.87) (Table 2).

**Table 1:** Radiographic scoring system for new bone formation in gout

**Sclerosis**	
**Definition**	Increased density of medullary or subcortical bone.
**Scoring rules**	The proximal and distal bones are scored separately for interphalangeal joints, metacarpophalangeal joints, and metatarsophalangeal joints (maximum score for individual joint is 6). Single bones are scored for the carpal and tarsal articulations (maximum score for individual bone is 3). For long bones, score the 1-cm distance from the articular surface. If the bone is eroded, score the available bone as the denominator.
**Joints scored**	The same bones scored in the gout-modified Sharp-van der Heijde scoring system for bone erosion (hand distal interphalangeal joints, hand proximal interphalangeal joints, interphalangeal joint of the thumb, metacarpophalangeal joints, base of the first metacarpal, multangular, scaphoid, lunate, radius, ulna, metatarsophalangeal joints, and interphalangeal joints of the hallux).
**Series and views**	Hand: anterior-posterior. Foot: anterior-posterior.
**Score**	0 = no sclerosis. 1 = < 1/3 bone affected. 2 = 1/3-2/3 bone affected. 3 = > 2/3 bone affected.
**Total score range**	0–276.
**Spur**	
**Definition**	A sharp spicule of dense bone proliferation extending at an acute angle from the cortex. This should be differentiated from an osteophyte, which arises along the joint margin.
**Scoring rules**	The proximal and distal bones are scored separately for interphalangeal joints, metacarpophalangeal joints, and metatarsophalangeal joints (maximum score for individual joint is 6). Single bones are scored for the carpal and tarsal articulations (maximum score for individual bone is 3). For long bones, score the 1-cm distance from the articular surface.
**Rules**	The same bones scored in the gout-modified Sharp-van der Heijde scoring system for bone erosion (hand distal interphalangeal joints, hand proximal interphalangeal joints, interphalangeal joint of the thumb, metacarpophalangeal joints, base of the first metacarpal, multangular, scaphoid, lunate, radius, ulna, metatarsophalangeal joints, and interphalangeal joints of the hallux).
**Series and views**	Hand: anterior-posterior and ball-catcher. Foot: anterior-posterior and oblique.
**Score**	0 = no spurs. 1 = 1 small spur. 2 = ≥ 2 small spurs and/or 1 moderate spur. 3 = ≥ 2 moderate spurs and/or ≥ 1 large spur(s).
**Total score range**	0–276.

**Table 2:** Inter-reader intraclass correlation coefficient (ICC) for the individual joints and hand and foot radiograph sets. For hand and foot radiograph sets, ICCs for baseline visit are shown

New bone formation feature	Individual joint analysis (***n*** = 80 joints)	Hand and foot radiograph sets (***n*** = 25 patients)
**Sclerosis**	0.84 (0.76–0.89)	0.94 (0.90–0.98)
**Spur**	0.81 (0.72–0.87)	0.76 (0.65–0.84)

### Comparison with CT measurements in individual joints

To assess construct validity, we compared the plain radiography scores with CT measurements of sclerosis and spur in individual joints. For sclerosis, the plain radiographic sclerosis score correlated with CT sclerosis ratio (*r* = 0.71, *P* < 0.001) and Hounsfield units (*r* = 0.74, *P* < 0.001). For joints with sclerosis on CT, the median (IQR) plain radiographic sclerosis score was 2 (1–2), compared with 0 (0–0) in joints without sclerosis (*P* < 0.001) (Table 3). For spur, the plain radiographic spur score correlated with CT spur length in millimeters (*r* = 0.65, *P* < 0.001), and spur ratio (*r* = 0.65, *P* < 0.001). For joints with spur on CT, the median (IQR) plain radiographic spur score was 1 (1–2), compared with 0 (0–0) in joints without spur (*P* < 0.001) (Table 3).

**Table 3:** Plain radiographic scores in individual joints according to computed tomography (CT) reference standard. Average plain radiographic scores for both readers were used in this analysis. Mann-Whitney *U* test *P* values are shown

	**Number of joints**	**Median (IQR) plain radiographic sclerosis score**	***P***
**CT sclerosis present**	34	2 (1–2)	< 0.001
**CT sclerosis absent**	46	0 (0–0)	
	**Number of joints**	**Median (IQR) plain radiographic spur score**	
**CT spur present**	19	1 (0–2)	< 0.001
**CT spur absent**	61	0 (0–0)	

*IQR* interquartile range

### Testing of the scoring system in sets of hand and foot radiographs

The scoring system was then tested in sets of hand and foot radiographs from 25 people with gout, obtained at baseline and after 2 years. Over the 2-year period, no significant differences were observed in sclerosis scores which changed from median (IQR) 8.5 (6–25) at baseline to 10 (5–25) at year 2, *P* = 0.076, or spur scores which changed from median (IQR) 4 (0–9) to 6 (1–8), *P* = 0.70 (Table 4). However, median (IQR) erosion scores increased from 24 (13–64) to 28 (18–70), *P* = 0.001.

**Table 4:** Change over time in radiographic parameters. Average plain radiographic scores for both readers were used in this analysis. Wilcoxon signed-rank test *P* values are shown

	Baseline	Year 2	***P***
**Sclerosis score, median (IQR)**	8.5 (5.5–25)	10 (5.25–8.25)	0.076
**Spur score, median (IQR)**	4.0 (0.25–8.5)	5.5 (0.75–8.25)	0.70
**Erosion score, median (IQR)**	24 (13–64)	28 (17.5–70)	0.001

*IQR* interquartile range

The inter-reader ICCs for the baseline radiographs were 0.94 (0.90–0.98) for sclerosis and 0.76 (0.65–0.84) for spur (Table 2). For the baseline radiographs, plain radiographic erosion scores correlated with sclerosis scores (for average of both readers, *r* = 0.87, *P* < 0.001) and spur scores (*r* = 0.71, *P* < 0.001) (Table 5). Sclerosis and spur scores also correlated (for average of both readers, *r* = 0.66, *P* < 0.001). Change in sclerosis and change in spur scores were correlated (*r* = 0.71, *P* < 0.001). However, change in erosion scores did not correlate with change in sclerosis scores (*r* = −0.04, *P* = 0.86) or change in spur scores (*r* = 0.15, *P* = 0.48).

**Table 5:** Spearman correlation coefficients for plain radiographic sclerosis, spur, and erosion total scores at baseline visit (*n* = 25) and for change over 2 years. Average plain radiographic scores for both readers were used in this analysis

		Baseline score		Year 2 scores		Change in scores	
		Sclerosis	Spur	Sclerosis	Spur	Sclerosis	Spur
**Erosion score**	*r*	0.87	0.71	0.88	0.68	−0.04	0.15
	*P*	< 0.001	< 0.001	< 0.001	< 0.001	0.86	0.48
**Sclerosis score**	*r*	−	0.66	−	0.66	−	0.71
	*P*	−	< 0.001	−	< 0.001	−	< 0.001

At the baseline visit, there was no association between new bone formation scores and gout disease duration or gout flare frequency (Supplementary Table 6). Spur scores correlated with serum urate at baseline (*r* = 0.41, *P* = 0.04), and change in spur score correlated with change in serum urate over the 2-year study period (*r* = 0.41, *P* = 0.04) (Supplementary Table 7).

## Discussion

This project has identified a feasible and reproducible method for scoring new bone formation features of sclerosis and spur in gout. This method correlates well with reference standard CT measurement and has good inter-reader reproducibility. This scoring system may facilitate research examining the mechanisms, impact, and treatment of structural bone lesions in gout, beyond bone erosion, which, with a few exceptions [1, 14], has been the major focus of research to date [15–18].

The literature review that informed the development of this scoring system showed substantial heterogeneity in radiological scoring systems for features of new bone formation both within and between different musculoskeletal diseases. In gout, some features of new bone formation are unique, and no published scoring system was easily transferable to gout. The initial scoring exercises demonstrated many of the challenges of scoring new bone formation and the need for clear definitions, standardization of projections, and reference images.

No previous plain radiographic new bone formation scoring systems have used a purely quantitative score. When this approach was tested for gout, inter-reader reliability was low, and this approach was also time-consuming. The time required for measurements and reliability may improve with the use of machine learning which has been demonstrated to reliably detect radiographic progression in axial spondyloarthropathy [19]. The project also showed the limitations of a single measurement of gout new bone formation features such as the proliferation score used in the PARS system; this method lacked inter-reader reproducibility when used in gout, and had low face validity for gout, as the features of new bone formation in gout are diverse and may not change in a consistent way. For this reason, we did not progress a single or composite scoring system following the initial scoring exercise.

Consistent with prior work [1, 3], this study suggests complex links between new bone formation and bone erosion in gout. The tophus has been strongly implicated in the development of bone erosion in gout [20–24], and prior research has shown associations between erosion, tophus, and new bone formation in gout [1], indicating that new bone formation may be repair phenomena triggered by the bone damage induced by the tophus. While the cross-sectional correlation analysis between erosion, spur, and sclerosis scores suggests that these lesions may reflect a generalized joint pathology, it is noteworthy that in the longitudinal analysis, change in erosion scores did not correlate with change in spur scores or change in sclerosis scores. These findings suggest that bone erosion and proliferative changes in gout may not be completely linked and that new bone formation scores may provide additional value to the assessment of structural bone disease in gout.

It is noteworthy that although there were small increases in bone erosion in the serial radiographs using the gout-modified Sharp-van der Heijde erosion score, no significant change in sclerosis or spur scores were observed over 2 years. These findings raise some uncertainty about the sensitivity to change of this scoring system, particularly noting that prior descriptive series have reported increased bone sclerosis within the affected joints in response to intensive urate-lowering therapy such as pegloticase [3, 4]. Importantly, participants in the longitudinal analysis were already established on urate-lowering therapy at the baseline assessment. Additional testing of the scoring system following initiation of urate-lowering therapy, following intensive urate-lowering, and particularly using randomized controlled clinical trial databases will be important to further understand the properties and performance of the scoring system. The future research agenda will also include testing whether new bone formation influences joint function, understanding the relationship between NBF and erosion healing in response to urate-lowering therapy, and whether other treatments such as anti-osteoclast therapy with zoledronate or denosumab influence new bone formation despite the lack of effect on erosion and joint space narrowing [16, 25]. Although we did observe an association between serum urate and spur scores over time, the study design did not allow us to determine the relationship between new bone formation scores and urate or inflammatory burden over the entire period of the disease (noting the mean disease duration was approximately 20 years); future studies should assess the cumulative burden of hyperuricemia and gout flares on new bone formation.

## Conclusion

In conclusion, a semi-quantitative plain radiographic scoring method for the assessment of bone sclerosis and spur in gout is feasible, valid, and reproducible. This method may facilitate consistent measurement of new bone formation in gout.

## Supplementary Information

**Additional file 1: Figure S1.** PRISMA flow diagram for literature review. **Table S1.** Frequency of the most commonly used scoring systems that assess any feature of new bone formation (n = 840 papers)^a^. Data are presented for the most frequently used radiographic scoring systems (> 5 articles). **Table S2.** Measurements for the first development exercise of individual joint analysis scoring. **Table S3.** Clinical features of the 20 participants in development exercise. Unless stated, data are presented as mean (SD). **Table S4.** Clinical features of the 25 participants with full scoring exercise. Unless stated, data are presented as mean (SD). **Table S5.** Intraclass correlation coefficient (ICC) for the first development exercise. ICC, intraclass correlation coefficient; CI, confidence interval; BLOKS, Boston Leeds Osteoarthritis Knee Score; PARS, Psoriatic Arthritis Ratingen Score. **Table S6.** Spearman correlation coefficients for baseline visit gout characteristics and new bone formation scores in the full scoring exercise. **Table S7.** Spearman correlation coefficients for changes in gout outcomes and new bone formation scores in the full scoring exercise over the Year 2 study period. Data are shown as Spearman correlation coefficient (P value).

## Data Availability

The data that supported the findings of this study are available on request from the corresponding author. The data are not publicly available due to privacy or ethical restrictions.

## Abbreviations

CI: Confidence intervals
CT: Computed tomography
ICC: Intraclass correlation coefficients
IP: Interphalangeal
IQR: Interquartile range
MSK: Musculoskeletal
MTP: Metatarsophalangeal
NBF: New bone formation
PARS: Proliferation score of the Psoriatic Arthritis Ratingen Score
SD: Standard deviation
TOMS: Thumb Base Osteoarthritis Magnetic Resonance Imaging Scoring System
