# Supplementary material for: Development of a radiographic scoring system for new bone formation in gout
Source: Arthritis Res Ther. 2021 Dec 8;23:296. doi: 10.1186/s13075-021-02683-9 (PMC8653557; doi:10.1186/s13075-021-02683-9)
Supplement: Supplementary file 1 — Additional file 1: Figure S1. PRISMA flow diagram for literature review. Table S1. Frequency of the most commonly used scoring systems that assess any feature of new bone formation (n = 840 papers)a. Data are presented for the most frequently used radiographic scoring systems (> 5 articles). Table S2. Measurements for the first development exercise of individual joint analysis scoring. Table S3. Clinical features of the 20 participants in development exercise. Unless stated, data are presented as mean (SD). Table S4. Clinical features of the 25 participants with full scoring exercise. Unless stated, data are presented as mean (SD). Table S5. Intraclass correlation coefficient (ICC) for the first development exercise. ICC, intraclass correlation coefficient; CI, confidence interval; BLOKS, Boston Leeds Osteoarthritis Knee Score; PARS, Psoriatic Arthritis Ratingen Score. Table S6. Spearman correlation coefficients for baseline visit gout characteristics and new bone formation scores in the full scoring exercise. Table S7. Spearman correlation coefficients for changes in gout outcomes and new bone formation scores in the full scoring exercise over the Year 2 study period. Data are shown as Spearman correlation coefficient (P value). [file 13075_2021_2683_MOESM1_ESM.zip › Supplementary data_clean.docx]

**SUPPLEMENTARY MATERIAL**

**Supplementary Table 1.** Frequency of the most commonly used scoring systems that assess any feature of new bone formation (n = 840 papers)^a^ . Data are presented for the most frequently used radiographic scoring systems (> 5 articles).

| **Name of scoring system** | **N (%)** | **Disease** | **Imaging Modality** | **NBF assessed as individual feature** |
| --- | --- | --- | --- | --- |
| Kellgren-Lawrence Radiographic Grading Criteria [1] | 431 (51.3%) | Osteoarthritis | Plain radiography | No |
| Whole Organ MRI Score (WORMS) [2] | 90 (10.7%) | Knee osteoarthritis | MRI | Yes |
| Modified New York Criteria [3] | 89 (10.6%) | Ankylosing spondylitis | Plain radiography | No |
| Modified Stoke Ankylosing Spondylitis Spine Score (mSASSS) [4] | 88 (10.5%) | Ankylosing Spondylitis | Plain radiography | No |
| Altman et al. [5] | 61 (7.3%) | Hip, hand & knee osteoarthritis | Plain radiography | Yes |
| Bath Ankylosing Spondylitis Radiology Index – Spine (BASRI-s) [6] | 43 (5.1%) | Ankylosing Spondylitis | Plain radiography | No |
| MRI Osteoarthritis Knee Score (MOAKS) [7] | 30 (3.6%) | Knee osteoarthritis | MRI | Yes |
| Boston-Leeds Osteoarthritis Knee Score (BLOKS) [8] | 15 (1.8%) | Knee osteoarthritis | MRI | Yes |
| Ahlblack et al. [9] | 14 (1.7%) | Knee osteoarthritis | Plain radiography | Yes |
| Ankylosing Spondylitis Spine MRI (ASspiMRI) score [10] | 14 (1.7%) | Ankylosing Spondylitis | MRI | No |
| Tonnis et al. [11] | 14 (1.7%) | Hip osteoarthritis | Plain radiography | No |
| Verbruggen et al. [12] | 12 (1.4%) | Hand osteoarthritis | Plain radiography | No |
| Glasgow Ultrasound Enthesitis Score (GUESS) [13] | 11 (1.3%) | Lower limb spondyloarthropathy | US | Yes |
| Brooker Score for Heterotopic Ossification [14] | 10 (1.2%) | Heterotopic ossification | Plain radiography | No |
| Knee Osteoarthritis Scoring System (KOSS) [15] | 10 (1.2%) | Knee osteoarthritis | MRI | Yes |
| Lane et al. [16] | 9 (1.1%) | Hand, hip & spine osteoarthritis | Plain radiography | Yes |
| Stoke Ankylosing Spondylitis Spine Score (SASSS) [17] | 8 (0.9%) | Ankylosing Spondylitis | MRI | No |
| Van Dijk et al. [18] | 8 (0.9%) | Ankle osteoarthritis | CT | No |
| Hand Osteoarthritis MRI Scoring System (HOAMRIS) [19] | 7 (0.8%) | Hand osteoarthritis | MRI | No |
| Croft et al. [20] | 6 (0.7%) | Hip osteoarthritis | Plain radiography | No |
| Kallman et al. [21] | 6 (0.7%) | Hand osteoarthritis | Plain radiography | Yes |
| Thumb base Osteoarthritis MRI Score (TOMS) [22] | 6 (0.7%) | Thumb base osteoarthritis | MRI | Yes |
| ^a^179 papers used two scoring methods, 23 papers used three scoring methods and 9 papers used four scoring methods. There were 63 further scoring systems that were identified. MRI, magnetic resonance imaging; US, ultrasound; CT, computed tomography; NBF, new bone formation. | | | | |

**Supplementary Table 2**. Measurements for the first development exercise of individual joint analysis scoring.

| **New bone formation feature** | **Tested measurements** |
| --- | --- |
| **Osteophyte** | 1. Length of largest osteophyte in mm 2. Ratio of the length of largest osteophyte relative to the width of the adjacent joint surface 3. Oslo Hand OA Score for osteophyte [23], proximal and distal bones scored separately: 0=none, 1=1-2 small osteophytes, 2=>2 small osteophytes or moderate osteophyte(s), 3=large osteophytes 4. BLOKS Knee Score for osteophyte [8], largest ostophyte within the region scored. The size of the osteophyte should reflect protuberance (how far the osteophyte extends from the joint rather than the total volume of the osteophyte). Score of 0-3: 0=none, 1=mild, 2=moderate, 3=severe. |
| **Sclerosis** | 1. Ratio of the depth of sclerosis relative to the width of the adjacent joint surface. Sclerosis measured transverse in long bone and perpendicular to the erosion or tophus if present 2. Degree of sclerosis expressed as a percentage of the entire joint affected. 3. Subjective sclerosis score from 0-3. 0=none, 1=mild, 2=moderate and 3=severe. |
| **Spur** | 1. Length of the largest spur in mm 2. Ratio of the length of the largest spur relative to the width of the adjacent joint surface 3. Composite score according to Stach et al. 2010. [24]: 0=no spur, 1=bone spur <1mm, 2=bone spur >1mm, 3=large widespread bone spurs 4. Oslo Hand OA Score adapted for spur (score 0-3) [23], proximal and distal bones scored separately: 0=none, 1=1-2 small spurs, 2=>2 small spurs or moderate spur(s), 3=large spurs 5. BLOKS Knee Score adapted for spur (score 0-3) [8]; largest spur within the region scored. The size of the spur should reflect protuberance (how far the spur extends from the joint rather than the total volume of the spur ). Score of 0-3: 0=none, 1=mild, 2=moderate, 3=severe. |
| **Periosteal new bone formation** | 1. Degree of periosteal reaction expressed as a ratio of the bone reaction relative to the length of the bone affected 2. Subjective score from 0-3. 0=none, 1=mild, 2=moderate and 3=severe. |
| **Ankylosis** | Present or absent |
| **Global** **New Bone Formation Score** | Total bone proliferation score (graded 0-4) based on the Psoriatic Arthritis Ratingen Score [25]. Composite score based of features including paraarticular spikes, supracortical bone formation, diaphysial thickening and enlargement of the bone compared to the opposite side or to the baseline radiographs Score of 0-4: 0=normal, 1=bony proliferation of 1-2mm or bone growth up to 25% of the original diameter, 2=bony proliferation of 2-3mm or bone growth up to 26-50% of the original diameter, 3= bony proliferation of >3mm or bone growth up to >50% of the original diameter, 4=bony ankylosis |
| BLOKS, Boston Leeds Osteoarthritis Knee Score; RAMRIS, Rheumatoid Arthritis Magnetic Resonance Imaging Scoring System; OA, osteoarthritis | |

**Supplementary Table 3. Clinical features of the 20 participants in development exercise.** Unless stated, data are presented as mean (SD).

| Age | 62 (12) years |
| --- | --- |
| Sex | 15 (75%) male, 5 (25%) female |
| Ethnicity | 11 NZ European, 4 Māori, 4 Pacific peoples, 1 Asian |
| Disease duration | 22 (15) years |
| Number of gout flares in the previous 6 months | 2.5 (3.1) |
| At least one subcutaneous tophus present | 12 (60%) |
| Allopurinol use, n (%) | 18 (90%) |
| Serum urate | 0.36 (0.10) mmol/L |
| Health Assessment Questionnaire-II | 0.5 (0.4) |

**Supplementary Table 4. Clinical features of the 25 participants with full scoring exercise.** Unless stated, data are presented as mean (SD).

|  | **Baseline** | **Year 2** |
| --- | --- | --- |
| Age | 57 (14) years | - |
| Male sex | 25 (100%) | - |
| Ethnicity | 13 Pacific peoples, 9 NZ European, 2 Māori, 1 Asian | - |
| Disease duration | 22 (11) years | - |
| Number of gout flares in the previous year | 10 (17) |  |
| Number of gout flares in the previous month | 0.84 (1.2) | 0.32 (0.6) |
| At least one subcutaneous tophus present | 18 (72%) | 18 (72%) |
| Allopurinol dose | 296 (149) mg/day | 430 (185) mg/day |
| Serum urate | 0.43 (0.11) mmol/L | 0.32 (0.06) mmol/L |
| Pain score (100mm visual analogue scale) | 2.0 (2.3) | 0.5 (1.3) |
| Health Assessment Questionnaire-II | 0.8 (0.6) | 0.3 (0.7) |
| Swollen joint count (/44) | 3 (6) | 1 (3) |
| Tender joint count (/44) | 3 (5) | 1 (2) |

**Supplementary** **Table 5.** Intraclass correlation coefficient (ICC) for the first development exercise.

| **New bone formation feature** | **Measurement** | **ICC (95% CI)** |
| --- | --- | --- |
| **Osteophyte** | Length of largest osteophyte in mm | 0.68 (0.55-0.78) |
|  | Osteophyte ratio | 0.67 (0.54-0.77) |
|  | Oslo proximal osteophyte | 0.23 (0.03-0.41) |
|  | Oslo distal osteophyte | 0.42 (0.23-0.57) |
|  | BLOKS osteophyte score | 0.66 (0.53-0.76) |
| **Sclerosis** | Sclerosis ratio | 0.42 (0.23-0.57) |
|  | Sclerosis percentage | 0.36 (0.16-0.52) |
|  | Sclerosis score | 0.54 (0.38-0.67) |
| **Spur** | Length of largest spur in mm | 0.28 (0.08-0.46) |
|  | Spur ratio | 0.34 (0.14-0.51) |
|  | Composite score | 0.31 (0.11-0.48) |
|  | Oslo proximal spur | 0.17 (-0.04-0.36) |
|  | Oslo distal spur | -0.05 (-0.25-0.15) |
|  | BLOKS spur score | 0.39 (0.20-0.55) |
| **Global assessment** | PARS | 0.64 (0.50-0.74) |

ICC, intraclass correlation coefficient; CI, confidence interval; BLOKS, Boston Leeds Osteoarthritis Knee Score; PARS, Psoriatic Arthritis Ratingen Score

**Supplementary Table 6. Spearman correlation coefficients for baseline visit gout characteristics and new bone formation scores in the full scoring exercise.**

| **Gout characteristic** |  | **Sclerosis score** | **Spur score** |
| --- | --- | --- | --- |
| Disease duration | *r* | 0.12 | -0.13 |
|  | p | 0.58 | 0.54 |
| Number of gout flares in the previous year | *r* | -0.16 | 0.20 |
|  | p | 0.44 | 0.33 |
| Serum urate | *r* | 0.37 | 0.41 |
|  | p | 0.07 | 0.04 |

**Supplementary Table 7. Spearman correlation coefficients for changes in gout outcomes and new bone formation scores in the full scoring exercise over the Year 2 study period.** Data are shown as Spearman correlation coefficient (P value).

|  |  | **Change in sclerosis score** | **Change in spur score** |
| --- | --- | --- | --- |
| Change in number of gout flares in the previous month | *r* | -0.09 | -0.16 |
|  | p | 0.68 | 0.44 |
| Change in serum urate | *r* | 0.26 | 0.41 |
|  | p | 0.21 | 0.04 |

**Supplementary references**

1. Kellgren JH, Lawrence JS. Radiological assessment of osteo-arthrosis. Ann Rheum Dis. 1957;16(4):494-502.

2. Peterfy CG, Guermazi A, Zaim S, Tirman PF, Miaux Y, White D, et al. Whole-Organ Magnetic Resonance Imaging Score (WORMS) of the knee in osteoarthritis. Osteoarthritis Cartilage. 2004;12(3):177-90.

3. Linden SVD, Valkenburg HA, Cats A. Evaluation of diagnostic criteria for ankylosing spondylitis. Arthritis Rheum. 1984;27(4):361-8.

4. Creemers MC, Franssen MJ, van't Hof MA, Gribnau FW, van de Putte LB, van Riel PL. Assessment of outcome in ankylosing spondylitis: an extended radiographic scoring system. Ann Rheum Dis. 2005;64(1):127-9.

5. Altman RD, Hochberg M, Murphy WA, Jr., Wolfe F, Lequesne M. Atlas of individual radiographic features in osteoarthritis. Osteoarthritis Cartilage. 1995;3(Suppl A):3-70.

6. MacKay K, Mack C, Brophy S, Calin A. The Bath Ankylosing Spondylitis Radiology Index (BASRI): a new, validated approach to disease assessment. Arthritis Rheum. 1998;41(12):2263-70.

7. Hunter DJ, Guermazi A, Lo GH, Grainger AJ, Conaghan PG, Boudreau RM, et al. Evolution of semi-quantitative whole joint assessment of knee OA: MOAKS (MRI Osteoarthritis Knee Score). Osteoarthritis Cartilage. 2011;19(8):990-1002.

8. Hunter DJ, Lo GH, Gale D, Grainger AJ, Guermazi A, Conaghan PG. The reliability of a new scoring system for knee osteoarthritis MRI and the validity of bone marrow lesion assessment: BLOKS (Boston Leeds Osteoarthritis Knee Score). Ann Rheum Dis. 2008;67(2):206-11.

9. Ahlback S. Osteoarthrosis of the knee. A radiographic investigation. Acta Radiol Diagn (Stockh). 1968:Suppl 277:7-72.

10. Braun J, Baraliakos X, Golder W, Brandt J, Rudwaleit M, Listing J, et al. Magnetic resonance imaging examinations of the spine in patients with ankylosing spondylitis, before and after successful therapy with infliximab: evaluation of a new scoring system. Arthritis Rheum. 2003;48(4):1126-36.

11. Tonnis D. Normal values of the hip joint for the evaluation of X-rays in children and adults. Clin Orthop Relat Res. 1976(119):39-47.

12. Verbruggen G, Veys EM. Numerical scoring systems for the anatomic evolution of osteoarthritis of the finger joints. Arthritis Rheum. 1996;39(2):308-20.

13. Balint PV, Kane D, Wilson H, McInnes IB, Sturrock RD. Ultrasonography of entheseal insertions in the lower limb in spondyloarthropathy. Ann Rheum Dis. 2002;61(10):905-10.

14. Brooker AF, Bowerman JW, Robinson RA, Riley LH, Jr. Ectopic ossification following total hip replacement. Incidence and a method of classification. J Bone Joint Surg Am. 1973;55(8):1629-32.

15. Kornaat PR, Ceulemans RY, Kroon HM, Riyazi N, Kloppenburg M, Carter WO, et al. MRI assessment of knee osteoarthritis: Knee Osteoarthritis Scoring System (KOSS)--inter-observer and intra-observer reproducibility of a compartment-based scoring system. Skeletal Radiol. 2005;34(2):95-102.

16. Lane NE, Nevitt MC, Genant HK, Hochberg MC. Reliability of new indices of radiographic osteoarthritis of the hand and hip and lumbar disc degeneration. J Rheumatol. 1993;20(11):1911-8.

17. Taylor HG, Wardle T, Beswick EJ, Dawes PT. The relationship of clinical and laboratory measurements to radiological change in ankylosing spondylitis. Br J Rheumatol. 1991;30(5):330-5.

18. van Dijk CN, Verhagen RA, Tol JL. Arthroscopy for problems after ankle fracture. J Bone Joint Surg Br. 1997;79(2):280-4.

19. Haugen IK, Eshed I, Gandjbakhch F, Foltz V, Ostergaard M, Boyesen P, et al. The longitudinal reliability and responsiveness of the OMERACT hand osteoarthritis magnetic resonance imaging scoring system (HOAMRIS). J Rheumatol. 2015;42(12):2486-91.

20. Croft P, Cooper C, Wickham C, Coggon D. Defining osteoarthritis of the hip for epidemiologic studies. Am J Epidemiol. 1990;132(3):514-22.

21. Kallman DA, Wigley FM, Scott WW, Jr., Hochberg MC, Tobin JD. New radiographic grading scales for osteoarthritis of the hand. Reliability for determining prevalence and progression. Arthritis Rheum. 1989;32(12):1584-91.

22. Kroon FPB, Conaghan PG, Foltz V, Gandjbakhch F, Peterfy C, Eshed I, et al. Development and reliability of the OMERACT thumb base osteoarthritis magnetic resonance imaging scoring system. J Rheumatol. 2017;44(11):1694-8.

23. Haugen IK, Lillegraven S, Slatkowsky-Christensen B, Haavardsholm EA, Sesseng S, Kvien TK, et al. Hand osteoarthritis and MRI: development and first validation step of the proposed Oslo Hand Osteoarthritis MRI score. Ann Rheum Dis. 2011;70(6):1033-8.

24. Stach CM, Bäuerle M, Englbrecht M, Kronke G, Engelke K, Manger B, et al. Periarticular bone structure in rheumatoid arthritis patients and healthy individuals assessed by high-resolution computed tomography. Arthritis Rheum. 2010;62(2):330-9.

25. Wassenberg S, Fischer-Kahle V, Herborn G, Rau R. A method to score radiographic change in psoriatic arthritis. Z Rheumatol. 2001;60(3):156-66.

**Supplementary figure legend**

**Supplementary Figure 1.** PRISMA flow diagram for literature review
