# Supplementary figures and images for: Development of a radiographic scoring system for new bone formation in gout
Source: Arthritis Res Ther. 2021 Dec 8;23:296. doi: 10.1186/s13075-021-02683-9 (PMC8653557; doi:10.1186/s13075-021-02683-9)

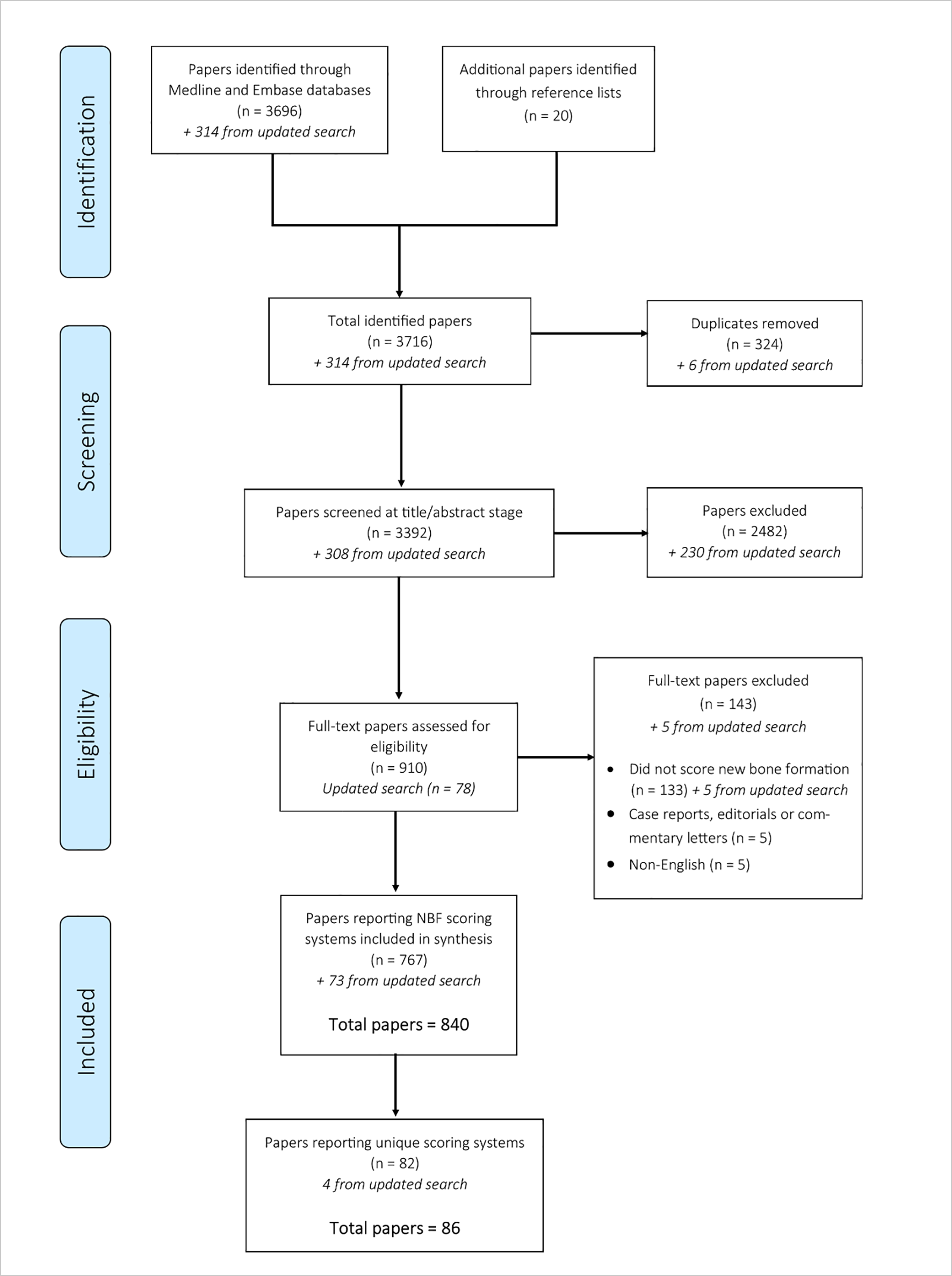

Supplement: Supplementary file 1 — Additional file 1: Figure S1. PRISMA flow diagram for literature review. Table S1. Frequency of the most commonly used scoring systems that assess any feature of new bone formation (n = 840 papers)a. Data are presented for the most frequently used radiographic scoring systems (> 5 articles). Table S2. Measurements for the first development exercise of individual joint analysis scoring. Table S3. Clinical features of the 20 participants in development exercise. Unless stated, data are presented as mean (SD). Table S4. Clinical features of the 25 participants with full scoring exercise. Unless stated, data are presented as mean (SD). Table S5. Intraclass correlation coefficient (ICC) for the first development exercise. ICC, intraclass correlation coefficient; CI, confidence interval; BLOKS, Boston Leeds Osteoarthritis Knee Score; PARS, Psoriatic Arthritis Ratingen Score. Table S6. Spearman correlation coefficients for baseline visit gout characteristics and new bone formation scores in the full scoring exercise. Table S7. Spearman correlation coefficients for changes in gout outcomes and new bone formation scores in the full scoring exercise over the Year 2 study period. Data are shown as Spearman correlation coefficient (P value). [file 13075_2021_2683_MOESM1_ESM.zip › Supplementary Figure 1.tif]
